# Design optimization of buttress type premium casing connection by modifying lower corner radius of stab flank

**DOI:** 10.1038/s41598-024-58881-3

**Published:** 2024-04-05

**Authors:** Byeongil Kim, Jong-Yun Yoon

**Affiliations:** 1 School of Mechanical Engineering, Yeungnam University, 280, Daehak-ro, Gyeongsan-si, Gyeongsang-buk-do 38541 Republic of Korea; 2 Department of Mechatronics Engineering, Incheon National University, 119 Academy-ro, Yeonsu-gu, Inchon, 22012 Republic of Korea

**Keywords:** Axial contacted stress, Buttress type, Design optimization, Premium casing connection, Lower corner radius of stab flank, Energy science and technology, Engineering, Materials science

## Abstract

Full-scale drilling for shale gas has been deepening, and the horizontal definitions have been increasing for drilling efficiency. Pressures and bending stresses are generated in the joints of gas-cored pipes, which seriously affect their durability. Premium casing connections are primarily forced at the interfaces of two parts, so the design of the threads is crucial, and the choice of optimal parameters is precisely related to their resistance to stresses. This article proposes a novel premium connection design, and its performance is validated through simulations to demonstrate good noise, vibration, and harshness (NVH) and durability performance. A parametric study is especially performed with the change of lower corner radius to observe maximum stress changes of the system and it is advised that a parameter be optimized. This study demonstrates that it is possible to design a premium connection capable of reducing the stress concentrated on the stab flank contact portion when drilling deep gas wells in response to parameter changes.

## Introduction

Owing to the lack of economic viability, it is difficult to extract the shale oil and gas contained in the shale layers; however, recent technological advancements have led an increased interest in them as a source of energy for the future. In 2005, the United States initiated shale gas exploration as a substitute energy source with the development of horizontal drilling and hydraulic fracturing technologies. Horizontal drilling is a technology, which increases mining productivity by increasing the surface area of gas fields and oil wells through the horizontal insertion of boreholes. The need for horizontal joining because the shale layer is placed deeper than the underlying sediment layer significantly increases the possibility that the pipe joints may deform. It is necessary to use a particular coupling with strong resistance to stress, fatigue, and high torque while drilling reasonably deep gas pockets and oil wells. This is called the ‘Premium Casing Connection’. In particular, the premium connection is subject to additional sealing to avoid leaks, and exhaustion performance is essential to tolerate the vibration caused by bending and torsion at the time of mining. The bottom hole pressure increases as the drilling depth increases, necessitating a change in the geometry of the connection element, such as the connecting thread deformation, screw pitch change, and shoulder and groove insertion, according to the American Petroleum Institute (API) standards. When a new connection type is created, it is anticipated to be advantageous for both the shale gas extraction and expedition of deep ocean.

Recently, various studies have focused on enhancing the design and performance of premium casing connections. The development level of gas and oil wells is largely dependent on the performance of the tubing equipment, and the fluidic tubing performance is largely determined by the connection components. Kim^1^ assessed the fluid connection system using mathematical modeling and field experiments. The pitch lengths of the screws were designed by Yamamoto et al.^2^; the contact stress centered on the threaded ends might be entirely diffused, thereby improving the performance of the bond, sealing, and abrasion resistance. Takano et al.^3^ improved the efficacy counter to tensile loading, pressure, and bending by negatively changing the loading flank angle. In addition, the correct choice of stab flank angle can improve the resistance to compression and optimize the radii of the corners to increase the wear resistance^3^. Xie and Tao^4^ conducted a computational study of the connection part based on the finite element method (FEM) in a thermal cycling situation, which is optimal for sealing, structural unity, and metal-to-metal sealing. Santi et al.^5^ developed a method for optimizing designs, which can increase the susceptibility of fractures only. A whole-scale fatigue experiment and stress concentration factor (SCF) analysis with cyclic loadings, fissure fracture areas, and FEM analysis were conducted^5^. Xie^6^ created the premium connection, JFEBEAR™, using the limit loading test with cyclic and thermal loading cycles. The premium connection is axisymmetric; hence, a 3D interpretation was not considered^6^.

Xie^7^ used the strain-rate model of material to offer a novel connection-making technique. It can accurately depict the features of a connection system under stress after makeup^7^. Stewart et al.^8^ developed a sliding friction testing device for conducting numerous sliding friction experiments on premium casing connections. The lubrication parameters, film thickness, and initial surface imperfections influence the observed coefficient of friction^8^. Because the current API standard premium connection design is unsuitable for gas and deeper wells, or severe drilling cases, Xu et al.^9^ created a novel casing system that stresses the sealability and tensile strength of joints. Galle et al.^10^ studied the modification of the constitutional properties when subjected to high tensile stresses with varying loading flank angles. A jump-out occurs when the plastic deformation exceeds the pin-thread height under increasing loading. The authors investigated whether the loading flange angle should be negative to prevent jump-out despite increased firing and attrition^10^. Xu et al.^11^ suggested a contact stress and loading analysis technique for premium systems with injection tubes based on the elastic dynamics. The relationship between the loading applied to the connected thread and average contact stress at the loading flank was computed using the thread surface geometry^11^. A method for determining the joint strength of API buttress thread teeth and premium connections was introduced by Xu et al.^12^. This method is founded on elastic mechanics and takes into account shoulder separation prevention and makeup torque^12^. It is demonstrated that the joint strength is significantly impacted by the ratio between the torque applied at the shoulder and the total torque. Specifically, as the ratio increases or decreases, the joint strength decreases. Hao^13^ redesigned the structure of the threaded portion of premium connection tubing and applied enhanced buttress threads of the casing to the tubing to improve connection performance and makeup performance.

Most recent technologies on the design of premium connection systems are concentrated on the sealing performance based on the contact stress distribution. In contrast to the prevalent finite element method, Xu and Yang^14^ investigated the sealability of premium connections and introduced a quantitative model that computes the gas sealing capacity of cone-to-cone premium connections directly. Gas sealing performance can be significantly enhanced by increasing both the axial sealing length and the radial sealing interference of the internal upset pipe, which possesses excellent sealing qualities. A novel approach was put forth by Yu et al.^15^ to assess the energy dissipation characteristics of premium connections in microslip shear layer mode. This technology addresses and enhances the shortcomings of current techniques for analyzing premium connections. Energy dissipation is more probable when the minimum microslip tangential force and the relative displacement of the contact surface are reduced in proportion to the stiffness of the shear layer. Moreover, the friction coefficient exerted a significantly greater influence on energy dissipation than the stiffness of the shear layer. Han and Fan^16^ investigated metal magnetic memory sensing for thread stress and ultrasonic phased array sensing for contact stress on the sealing surface. The tendency of the tangential component of the magnetic field intensity to vary is identical to that of the stress at the threaded connection, which is directly proportional to the variation in the metal-to-metal seal contact stress. In contrast to the static analysis method that was previously utilized to assess the sealing performance of premium connections, Yu et al.^17^ introduced a novel approach which developed a dynamic model of the premium connection's sealing surface in accordance with the vibration equation of the elastic rod. The analysis focused on the hysteresis properties and energy dissipation mechanism of the sealing surface. The effectiveness of energy dissipation theory in analyzing the sealing performance of premium connections under dynamic loading is demonstrated to be superior to that of conventional static contact analysis.

In their semi-theoretical model, Xu et al.^18^ attempted to quantify the leakage rate of premium connections. Roughness and geometric mean contact pressure for elastic–plastic contact of axisymmetric sinusoidal micro-convex bodies using actual area prediction formulas. In light of the irregular distribution of contact pressure between the sealing surfaces, the precise micro-leakage rate of the premium connection was calculated. In their investigation into the sealability of premium connections, Yu et al.^19^ utilized the fractal function to define the rough profile. Additionally, they examined the impact of fractal parameters on the contact behavior of surfaces that are rugged^19^. After analyzing Von Mises stress and contact pressure with a FEM-based model, the sealability was assessed by applying contact strength theory. Li et al.^20^ investigated Von Mises stress and contact pressure distributions in a variety of scenarios using finite element analysis and ISO 13679, which are extensively employed to assess the performance of tube and casing connections^20^. The influence of bending load on Von Mises stress distribution and contact pressure was discovered to be substantial. Finite element simulation has the capability to accurately replicate ISO 13679 test procedures, generate distributions of stress and contact pressure, and function as a benchmark for assessing the performance of premium connections. Yang et al.^21^ computed the elastic–plastic contact pressure distribution at the interface of sealing by integrating the von Mises yield criterion and Hertz contact pressure. The gas–gas sealing capacity was also deduced using the sealing criterion derived from Murtagian's experimental findings. The study employed the suggested framework to assess and contrast the impact of supplementary makeup torque at the interface of the seal on critical sealability indicators—contact width, yield width, average contact pressure, and gas seal capacity—as well as the distribution of seal contact pressure^21^. The findings demonstrate a strong correlation between the additional makeup torque applied at the sealing interface and the variations in gas sealing capacity and sealability parameters.

Evidently, majority of the pipe connection system research focuses on the loading-free components, such as the thread section of the premium connection's loading flange angle under tensile loading, sealability, and integrity. The discharge of oil and shale gas would result in both environmental pollution and financial loss when the oil and gas definitions are formed. To minimize leakage, the sealing performance should be maximized. However, in the design of premium connections, parameter changes related to the piercing-free component may also influence the ability of the threaded component to seal. Sophisticated loading is required to determine the stress properties in the sludge connection to guarantee optimum sealing performance. Furthermore, only a few local and foreign studies have focused on the flank-related factors. Thus, this study aims to understands the durability and vibration properties of the premium casing system regarding the parameters connected to the piercing-free region of the thread, and offers the best design path based on the parametric analysis.

Starting from the buttress-type API 5 B model, study aims to investigate the variation in the durability of the threaded connections from the vibrations owing to the change in the radius of the lower corner in the stab flank and contrast the differences. Because the analytical results from the three-dimensional and axisymmetric models are often the same ("Background theory"), this study uses the 2D axisymmetric model. Although this model is easier to understand than the 3D-based model and shows the benefit of accelerating the study, a 3D analysis is still required to fully understand the scenarios, in which the bending loadings are present.

This paper is organized as follows. "Background theory" discusses the background theory on the standard threaded connection system, especially for the stress calculation. “Modeling and Preprocessing” describes the computer aided modeling, procedure to change the thread characteristics, such as the stab flank, procedure to choose a mesh, which is best for threaded connection components, and procedure to adjust the coefficient of friction between the threaded contact parts. An analytical approach using the ANSYS™ is presented in "Parametric study for optimized design". The findings of this study and an examination of vibration and durability changes as a consequence of parameter adjustments are discussed in "Results". The conclusions and future goals are discussed "Conclusions".

## Background theory

**Figure 1:**
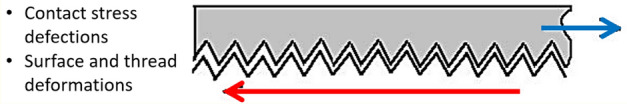
Micro effects of torque on threaded connection systems.

Gaining insight into the significance of friction in both the underhead and threaded contact zones is crucial for delineating the correlation between tension, angle, and torque. Hundreds of variables can influence the tension that is produced in a threaded connection when a tightening torque is applied. Fortunately, torque–angle signature curves for the majority of mounted joints are obtainable. A practical comprehension of the engineering mechanics of threaded connections and the integration of torque–angle curves with a few straightforward calculations enable one to acquire the knowledge required to assess the attributes of specific connection tightening processes^22–24^. When prevailing torque locking features are implemented, an additional prevailing torque zone is incorporated into the model. To put it more broadly, the prevailing torque may be caused by frictional drag on the shank or threads as a result of misaligned components, chips, or other extraneous material in the threads; or, inadvertent interference from out-of-tolerance threads. Surface and thread deformations, in addition to contact stress defections of plating and coatings, are among the micro effects. The aforementioned effects are depicted in Fig. 1.

As a practicable starting point for all threaded fastener tightening analyses, estimate the relative magnitudes of torque and clamp force using the fundamental elastic torque-tension equation. One can formulate models for the tightening process by commencing with the equation that establishes a linear correlation between tension and torque:

1 $$T=KDF$$

where T is a torque, K is a nut factor, D is a nominal diameter, and F is a force. By the following formula, deformation of the fastener and angle of turn are geometrically related.

2 $$\delta =\frac{\alpha }{360}P$$

Here, $\delta$ represents the deformation, $\alpha$ represents the angle, and P represents the pitch of thread. For a fastener to be correlated directly with the stress-induced total strain in the fastener, it must be tightened on an infinitely rigid joint. It has been demonstrated through extensive experimentation that the tension generated is directly proportional to the angle of rotation from the elastic origin. By projecting a line tangent to the elastic portion of the torque–angle curve in the opposite direction of zero torque, the elastic origin can be determined. The sum of the compression of the confined components and the extension of the fastener constitutes the total angle of turn. The following equation defines the fundamental relationship between tension and strain in the elastic region.

3 $$\sigma =\varepsilon E$$

Here, $\sigma$ is the stress, $\varepsilon$ is the strain, and E is the Young’s modulus of a certain material. The calculation of the strain of a tension-loaded fastener or metal rod is performed utilizing the subsequent equation, where ${L}_{e}$ and A stand for the effective length and the cross-sectional area.

4 $$\Delta =\frac{F{L}_{e}}{AE}$$

In order to determine clamping load using the turn-to-tension method, both the bolt spring rate and the spring rate of the clamped components must be known. This is due to the fact that turning the bolt causes the fastener to extend, while simultaneously compressing the components under clamping. Using the following equation, one can express the slope of the force–angle of turn relationship.

5 $$\frac{\Delta F}{\Delta\Theta }=\left(\frac{{K}_{B}{K}_{C}}{{K}_{B}+{K}_{C}}\right)\frac{P}{360}$$

Here ${K}_{B}$ and ${K}_{C}$ represent the bolt and joint spring rate. The relationship that results from taking the first derivative of the fundamental equation concerning torque is as follows:

6 $$\Delta T=KD\Delta F, \Delta F=\frac{\Delta T}{KD}$$

By replacing $\Delta F$ with the given value in the force–angle of turn equation, one obtains the torque–angle slope equation, which can be utilized to approximate the spring rate of mounted joints:

7 $$\frac{\Delta T}{\Delta\Theta }=\left(\frac{{K}_{B}{K}_{C}}{{K}_{B}+{K}_{C}}\right)\frac{PKD}{360}$$

The equation provided is utilized to estimate the spring rate of the fastener:

8 $${K}_{B}=\frac{F}{\Delta }\frac{LB}{\varepsilon }=\frac{AE}{{L}_{e}}$$

Following this, $\Delta T/\Delta\Theta$, the slope of the elastic clamping region of the torque–angle curve, is calculated using the curve as a reference. By assuming a value for *K*, the spring rate of the joint can be determined in the subsequent manner:

9 $${K}_{C}=\frac{\Delta T/\Delta\Theta }{\frac{KDP}{360}{K}_{B}-\frac{\Delta T}{\Delta\Theta }}{K}_{B}$$

The equivalent contact radius is determined by the pressure distribution and geometric parameters of the thread. Variations in frictional torques can be attributed to the impact of pressure distribution on the effective radius of the bearing surface caused by distinct fastener support surfaces. Geometric variations in the threads give rise to alterations in the contact pressure and friction radius at both the thread surface and the end surface of the screw head. These modifications influence the magnitude of the friction torque and induce a distribution of the preload force across multiple bolts. For the purpose of conducting a more comprehensive analysis of the stress distribution within the precision threaded connection structure, we model the threaded tooth as a cantilever beam and the force acting on the threaded tooth as a concentrated force denoted as ${F}_{x}$. This simplifies the expression for the stress distribution in the threaded connection:

10 $$\sigma =\frac{{F}_{x}}{A}$$

The axial load at any given section is then:

11 $${F}_{x}=F\frac{{\text{sinh}}x\lambda }{{\text{sinh}}L\lambda }$$

where $\lambda$ is a parameter about the thread structure, related to the geometric parameters of the thread. As shown in Eq. (7), when it changes, the axial load component ${F}_{x}$ at any cross-section in the thread also changes, influencing the stress distribution on the connection cross-section. As a result, the assembly tension distribution is complex and irregular along the screw axis at various screw connection positions when a thread manufacturing error occurs. The dispersion of the precision thread preload is influenced by the equivalent contact radius that varies during the tightening process when there is a geometric deviation between various bolts, as determined by the analysis above. Simultaneously, throughout an engagement, the concentrated force on the thread varies, which exacerbates the degree of irregular contact stress distribution on the threaded connection structure. We do not consider the friction coefficient of the contact surface in this context, as it is dependent on the material of the component.

While it is possible to theoretically approximate the stress in threaded connection systems featuring simple shapes like the one described, the theoretical foundation for relatively complex shapes, such as the buttress type utilized in premium connection systems, is not well understood. As a result, this study proposes a finite element analysis-based stress analysis methodology for parametric studies and optimal designs.

## Modeling and preprocessing

**Figure 2:**
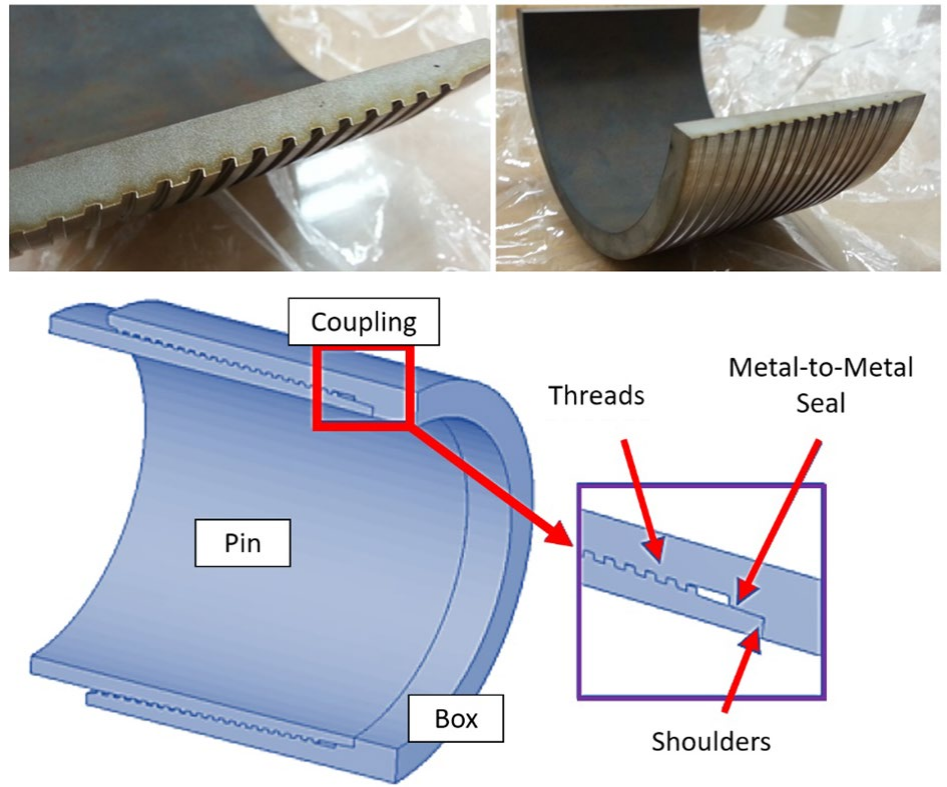
Detailed segments of premium casing system.

Because the two-dimensional (2D) axisymmetric model is substantially plainer than the three-dimensional (3D) model and analysis time is not noticeably longer, it is employed for the analysis of the premium casing system. The analysis findings of the 3D-based and 2D-based axisymmetric models are in excellent agreement with respect to the tensile and compressive forces; however, an analysis based on 3D modeling is necessary to examine the bending^25^. Figure 2 shows a sample of premium connection system and detailed segments of premium casing system, which is the object of this article.

### 2D axisymmetric model

**Figure 3:**
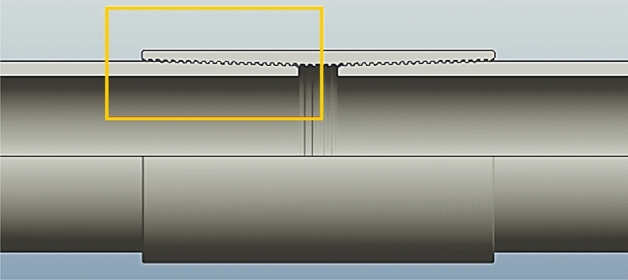
Typical pipe casing connection and its cross-section.

**Figure 4:**
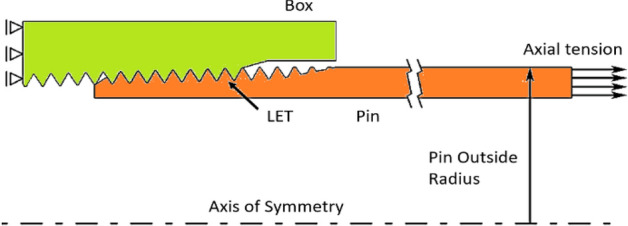
Axisymmetric 2D model used for structural analysis.

A section of a typical pipe connection and corresponding axisymmetric 2D model used for the structural analysis are shown in Figs. 3 and 4.

**Figure 5:**
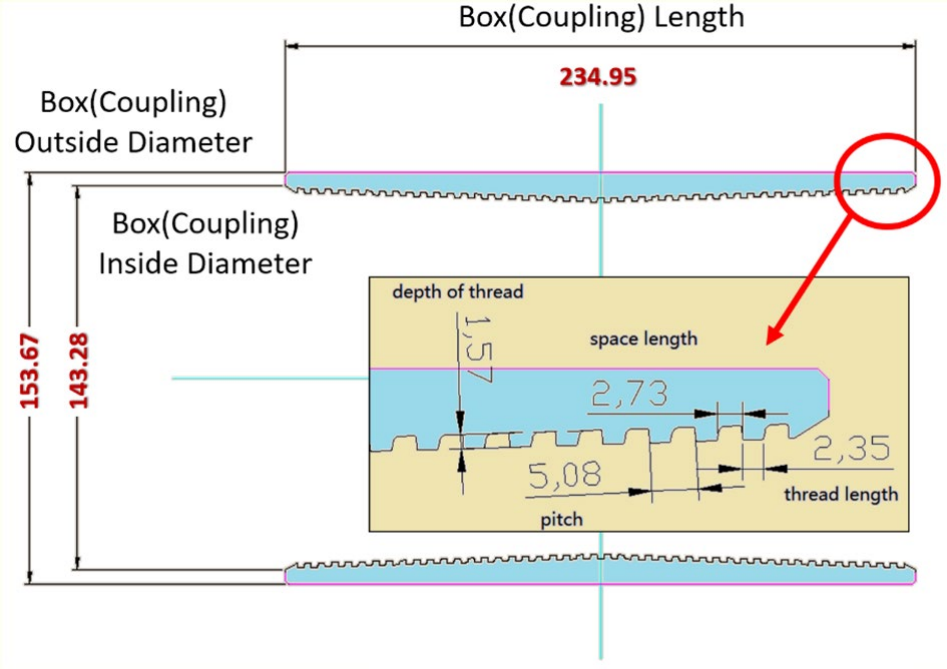
Geometry of typical connection (API standard): box (coupling).

**Figure 6:**
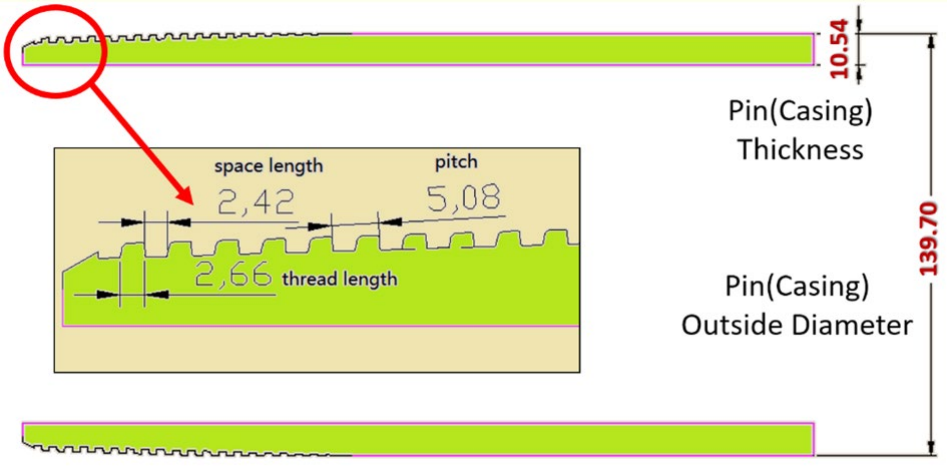
Geometry of typical connection (API standard): pin (casing).

**Figure 7:**
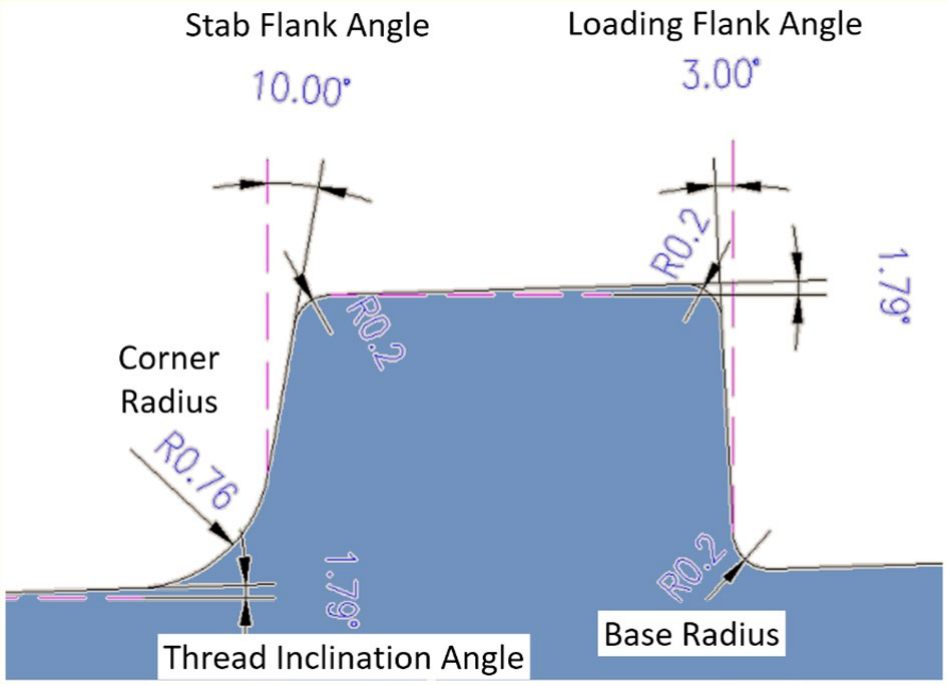
Thread geometry.

The box, pin, and thread geometries of the API premium connection system are shown in Figs. 5, 6 and 7. The settings applied to the box components are shown in Fig. 5. The box is 234.95 mm long with an outer diameter of 153.67 mm, and inside diameter of 143.26 mm. The box part is extended to create geometrical thread parameters. The pitch is 5.08 mm, thread length is 2.35 mm, space length is 2.73 mm, and thread thickness is 1.57 mm. The parameters connected to the pins are shown in Fig. 6. The pin has an outer diameter of 139.70 mm and thickness of 10.54 mm. Part of the pin thread is also expanded, and geometric specifications of the thread are compiled. The spacing is 2.42 mm long, pitch is 5.08 mm, and thread is 2.66 mm long.

The thread-related parameters are shown in Fig. 7. The top corner of the stab flank has a radius of 0.2 mm and bottom corner of the loading flank had a radius of 0.2 mm. The loading flank radii at the upper right corner also have the same dimension of 0.2 mm.

### Finite element (FE) model: optimizing mesh size

**Figure 8:**
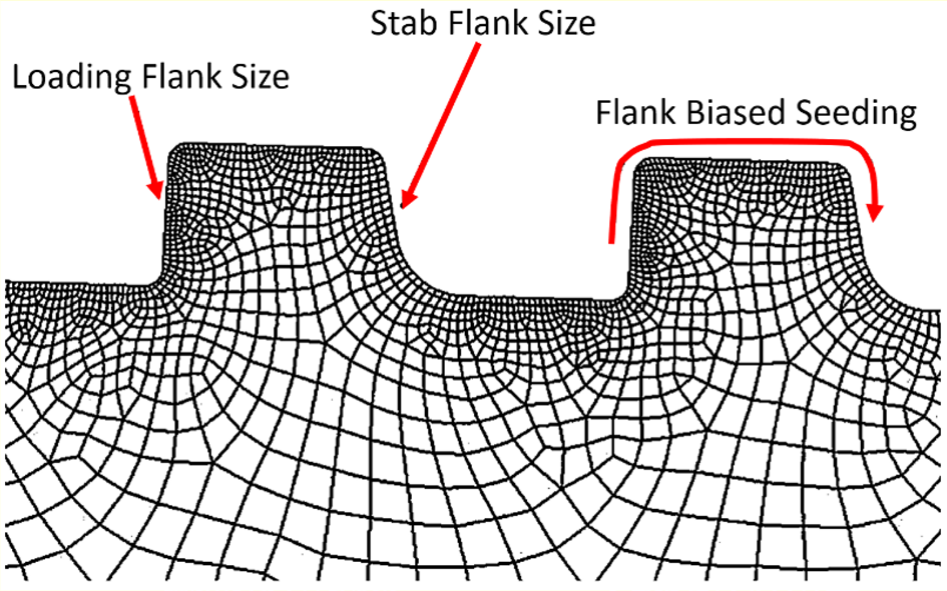
Mesh structure for structural analysis.

After creating the design file (computer-aided design) for studying the premium connection system, finite element modeling is used to create the mesh. The global and local sizes, size of the loading and stab flank, and count of the flank elements are the five factors that determine the FE mesh, as described in Fig. 8. In this 2D study, quad elements are primarily used.

**Figure 9:**
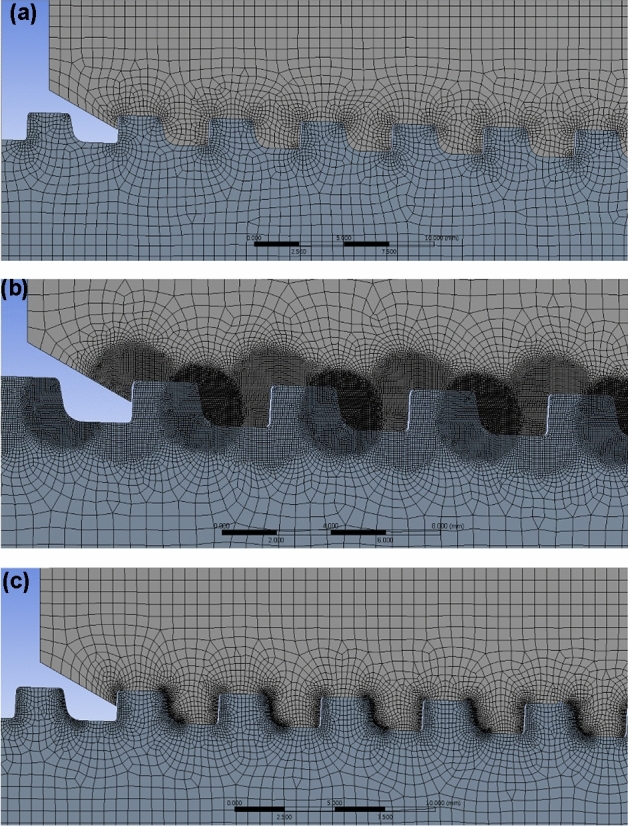
FE modeling with (**a**) coarse, (**b**) round, (**c**) line mesh.

The mesh is modeled using a coarse mesh whose size is considerably big for minimizing the analysis time at the highest estimate, as illustrated in Fig. 9a. The FE model is first created with a 0.6 mm size global mesh, and then, for the section of the thread, at which most stress is focused, a mesh is created with a 0.1 mm size local mesh.

The mesh may be described as a non-piercing component for an accurate measurement of the stress distribution. To apply the contact status, the perforation size of the pin portion must be smaller than that of the box. The local element sizes of the thread at pin with round mesh, as shown in Fig. 9b, are listed here. For the pin part, the element size at the loading flank is 0.05 mm, compared to the 0.03 mm of the stab flank. For the box part, the element size at the stab flank is 0.05 mm, compared to the 0.08 mm for the loading flank. The round mesh has the disadvantage of increasing the analysis duration by producing overly thin elements for the thread boundary, where the von Mises stress is focused, and in the region where a thorough analysis is not necessary.

To evaluate the stress distribution accurately, the mesh may be referred to as the non-piercing component. The pin section must have a hole smaller than the coupling (box) to maintain the contact status. The local element sizes in the threaded section of the pin with line mesh, as shown in Fig. 9c, are listed here. The element size of the loading flank at the pin is 0.05 mm, compared to the 0.03 mm for the stab flank. The element size at the stab flank at coupling is 0.05 mm, while that at the loading flank is 0.08 mm. By creating excessively small meshes for the thread boundary, where the real stress is focused and where a detailed analysis is not required, the round mesh method shows a defect in lengthening computation times.

### Elapsed time for analysis

An optimal mesh model must be selected to perform the necessary tensile and compressive assessments for this study. For the optimization study, the finely generated round, line, and standard coarse elements are compared, focusing on the time and results from the analysis focused on the concentrated stress at the pin last engaged thread (LET) section for the premium casing system.

**Figure 10:**
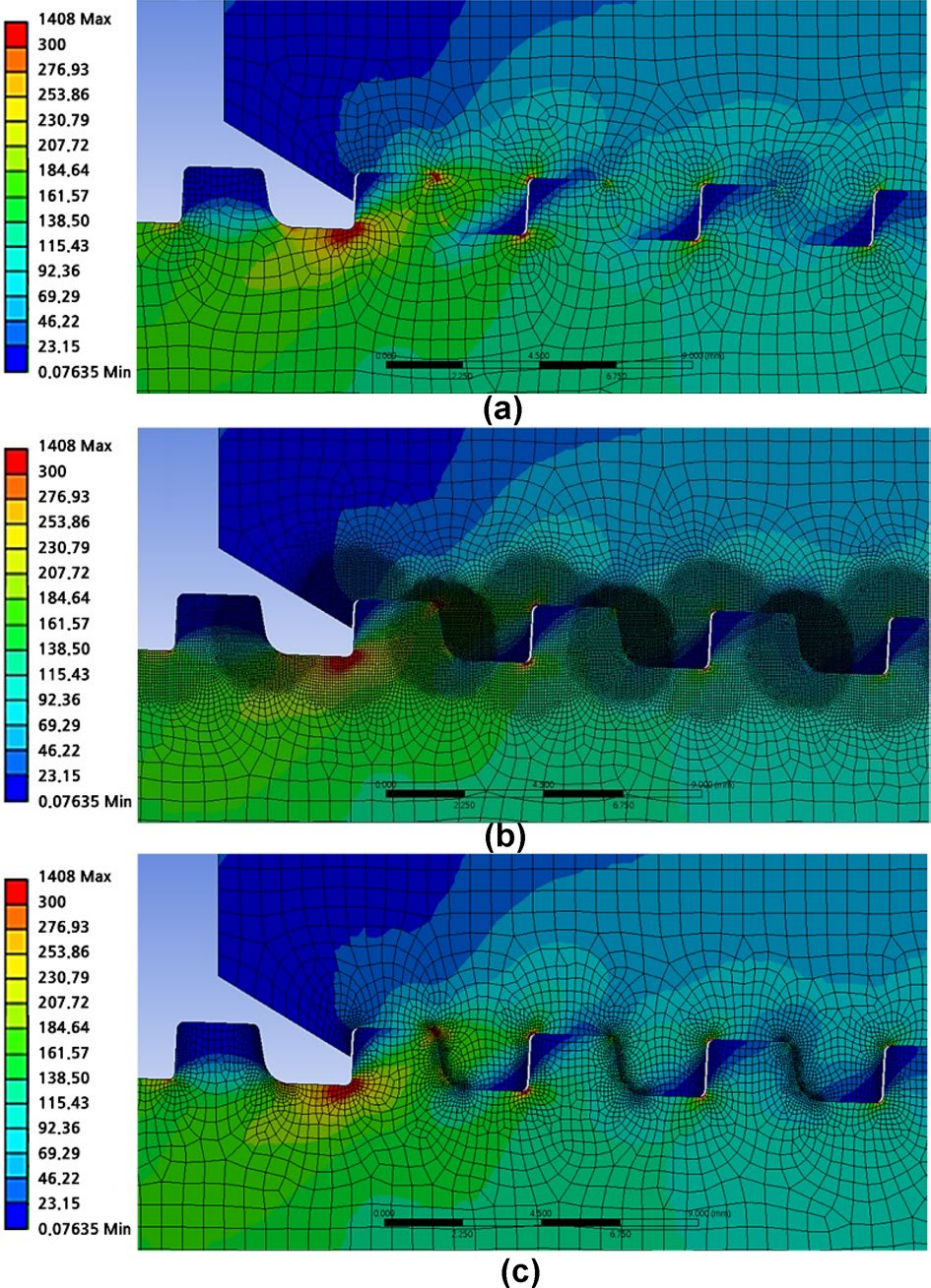
Analysis results comparison with (**a**) coarse, (**b**) round, (**c**) line mesh (LET part).

As shown in Fig. 10, there are no appreciable variations in the contours for stress with the three different FE models. Table 1 compares the amount of time needed to create and analyze the different mesh sizes, with the coarse mesh requiring the least amount of time. For the round and line meshes, using a coarse mesh lengthened the analysis durations by 5.3 and 2.9 times, respectively. The time required is determined based on the performance of the computer and other elements. The mesh-generation approach is selected to produce a coarse mesh to obtain the same outcome in the least amount of time.

**Table 1:** Time for generation and analysis with each type of element.

Type of mesh	Generation time (s)	Analysis time (s)	Total time (s)
Coarse	54	75	129
Round	288	396	684 (530%↑)
Line	174	201	375 (290%↑)

### Friction coefficient

The study should consider the friction coefficient between the fastener and box. Lubrication is applied when a joint is used, which requires a coefficient of friction. It seems reasonable to set the coefficient of friction to 0.05 in the Mortensen's^26^ examination of the bolt-on friction analysis.

## Parametric study for optimized design

As previously mentioned, the premium casing system serves as a component, which joins the flow pipe; thus, it is essential to create an optimum thread because the tension and compressive force are important factors in both the installation and usage. Thread stress analysis is necessary to optimize the design, and the best dimensions must be determined via parametric analysis using a range of parameters.

**Figure 11:**
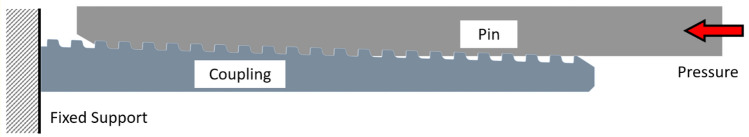
Initial and boundary conditions.

**Figure 12:**
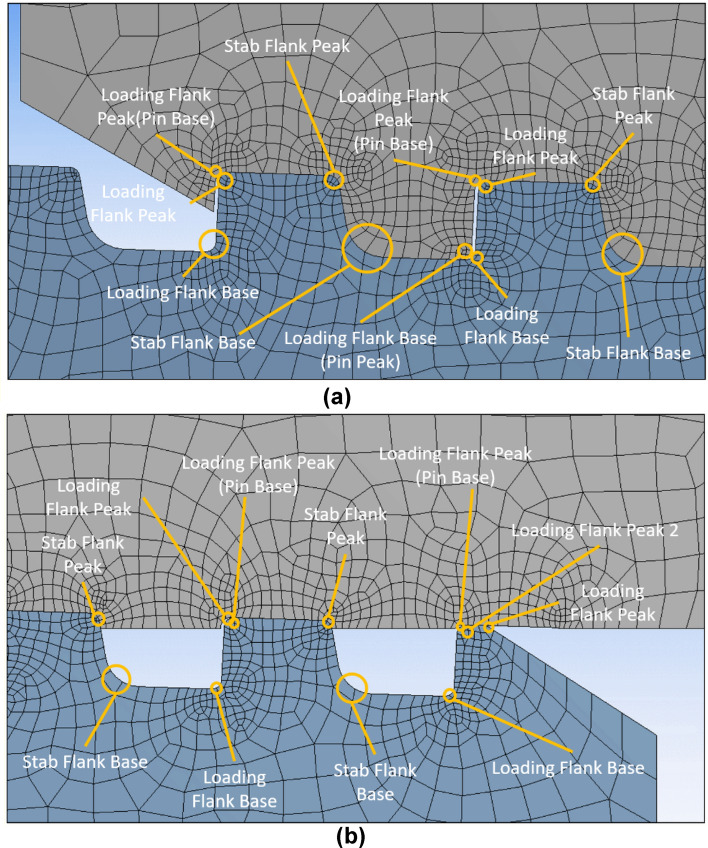
Name of each part: (**a**) pin LET, (**b**) coupling LET.

The analysis is based on the von Mises stress of the premium connection system and buttress-type API 5 B basic model. Changes in both the attributes and size are systematically examined. It is assumed that the premium casing connection is subjected to the compression force associated with the most strained location when contact occurs between the pin and coupling stab flange. The initial value condition and boundary conditions for the finite element numerical simulations are as follows: (1) the leftmost plane of the coupling has a fixed support, (2) 150 MPa pressure is applied on the rightmost plane of the pin to the left, and (3) the upper plane of the pin and the lower plane of the coupling are not allowed to move in the vertical direction, as described in Fig. 11. Three separate points are selected for each component, as shown in Fig. 12, and the stress in each part is recorded and investigated.

Figure 12a shows the identification of each component in relation to the LET section of the pin. The lower corner sections of "Pin Thread # 1 (Pin LET)" are home to the "Loading Flank Base" and "Stab Flank Base", while the top corner portions refer to the "Loading Flank Peak (Pin Base)" and "Loading Flank Peak". In the top corner of the "Pin Thread # 2", "Stab Flank Peak", "Loading Flank Peak", and "Loading Flank Peak" are all similarly referred to. Figure 12b shows the names of each component related to the LET component of coupling. As can be seen in the picture, "Loading Flank Peak (Pin Base)", "Loading Flank Peak", and "Loading Flank Peak 2" are designated as the three locations in the top corner of the "Coupling Thread # 1 (LET)." There is just one site selected for the 'Flank Base'. 'Coupling Thread #2's' top corner is designated for the 'Loading Thread Flank Peak (Pin Base)', ‘Loading Flank Peak’, and 'Stab Flank Peak’, while the bottom corner is designated for the 'Loading Flank Base' and 'Stab Flank Base.’ Only one "Stab Flank Peak" and one "Stab Flank Base" are assigned for "Coupling Thread #3" in the upper and lower corner portions, respectively.

**Figure 13:**
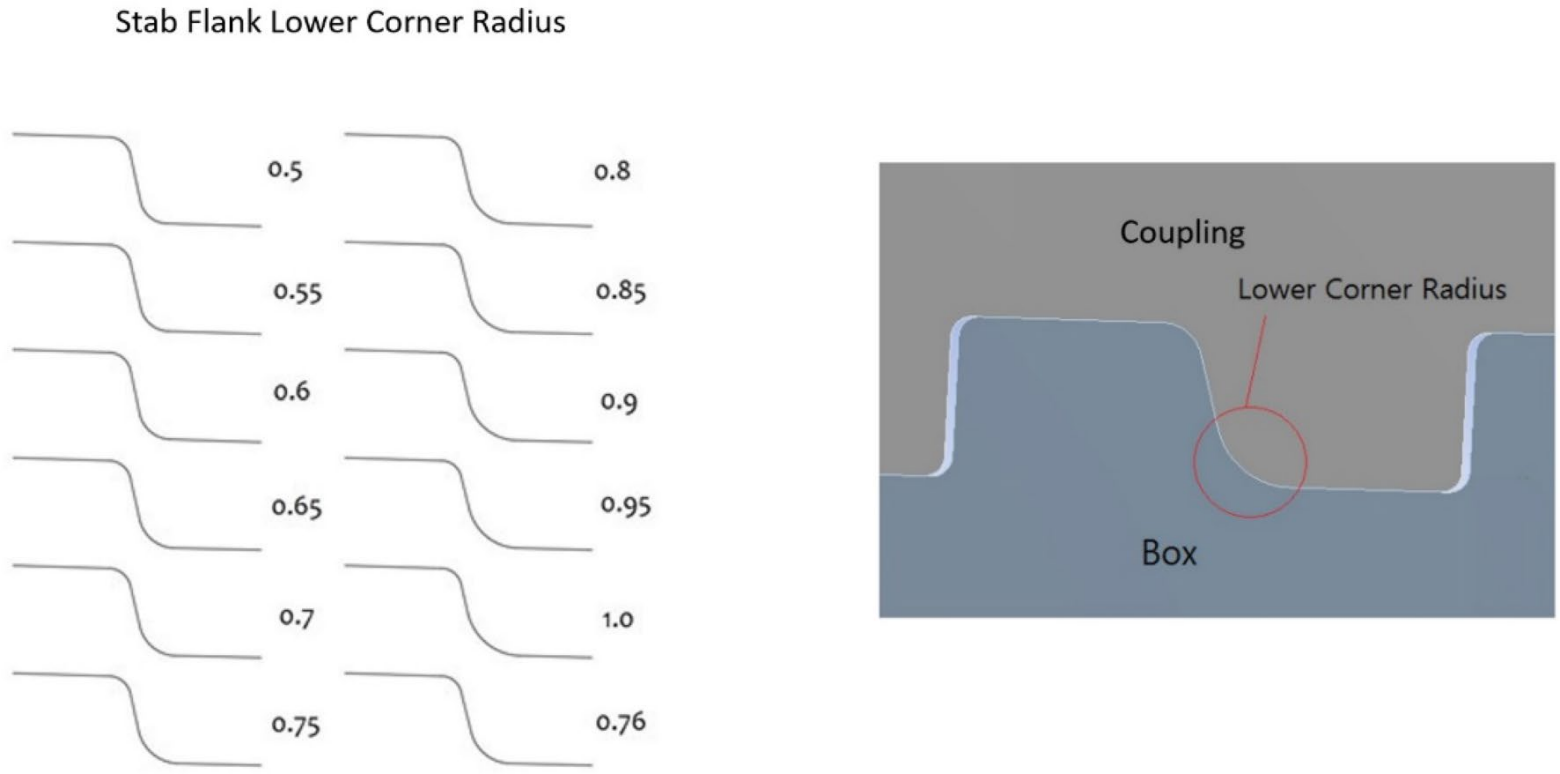
Stab flank lower corner radius (increased from 0.5 to 1.0 cm, 0.05 cm interval).

Because the previous research showed that the corner section receives the bulk of the concentrated stress, the stab flank's angle was fixed to 13° and radius at the upper corner as 0.40 cm to obtain a better outcome. Accordingly, the radius of the bottom corner changed. Initially, the radius of the bottom corner was 0.76 cm. To avoid deviating from the conventional rule and to investigate the deviation of axial stress, the radius at the lower corner of the stab flank is increased from 0.50 to 1.00 cm in steps of 0.05 cm, as illustrated in Fig. 13.

### Coupling thread 1 (coupling LET)

**Figure 14:**
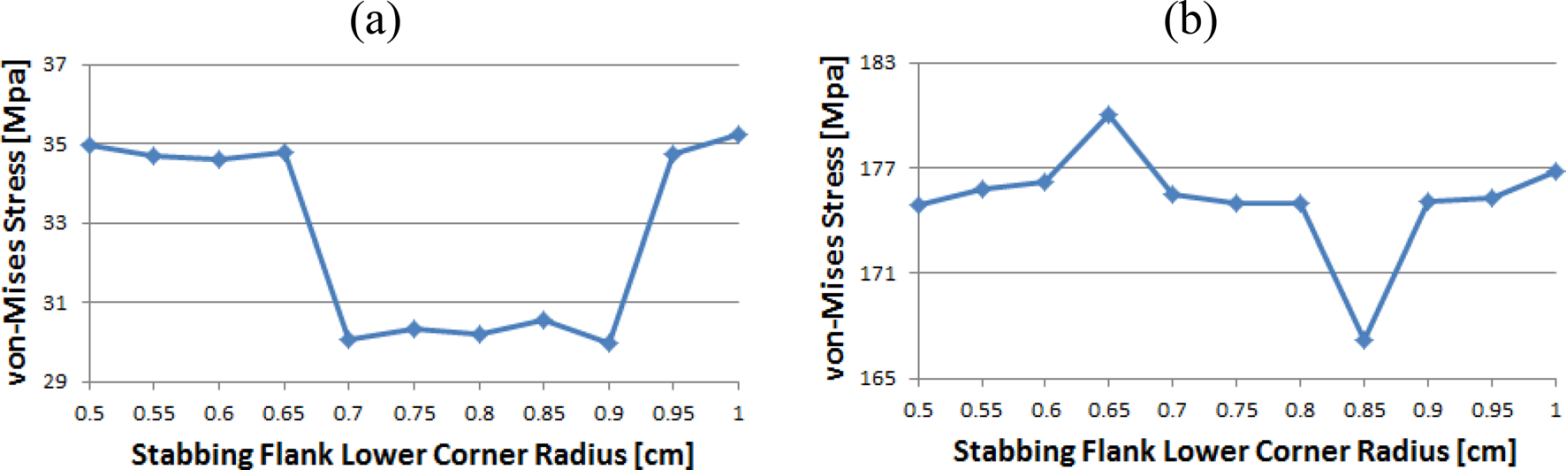
Stress change trend with lower corner radius of stab flank: (**a**) loading flank peak 2, (**b**) loading flank peak (pin base) of coupling thread 1 (coupling LET).

Figure 14 shows the instance of Loading Flank Peak 2, and the stress values are initially near 35 MPa from the radius of 0.5–0.65 without a significant difference; however, with 0.7 onwards, the values decreased to near 30 MPa. The value increased again from 0.95. The maximum and minimum values are determined to be at 1 and 0.9, respectively. The maximum value of the loading flank peak (pin base) can be seen at 0.65, increasing continuously from the beginning. Beginning at 0.7, the value decreased until it reached a minimum value of 0.85. From 0.9, the value increased significantly.

**Figure 15:**
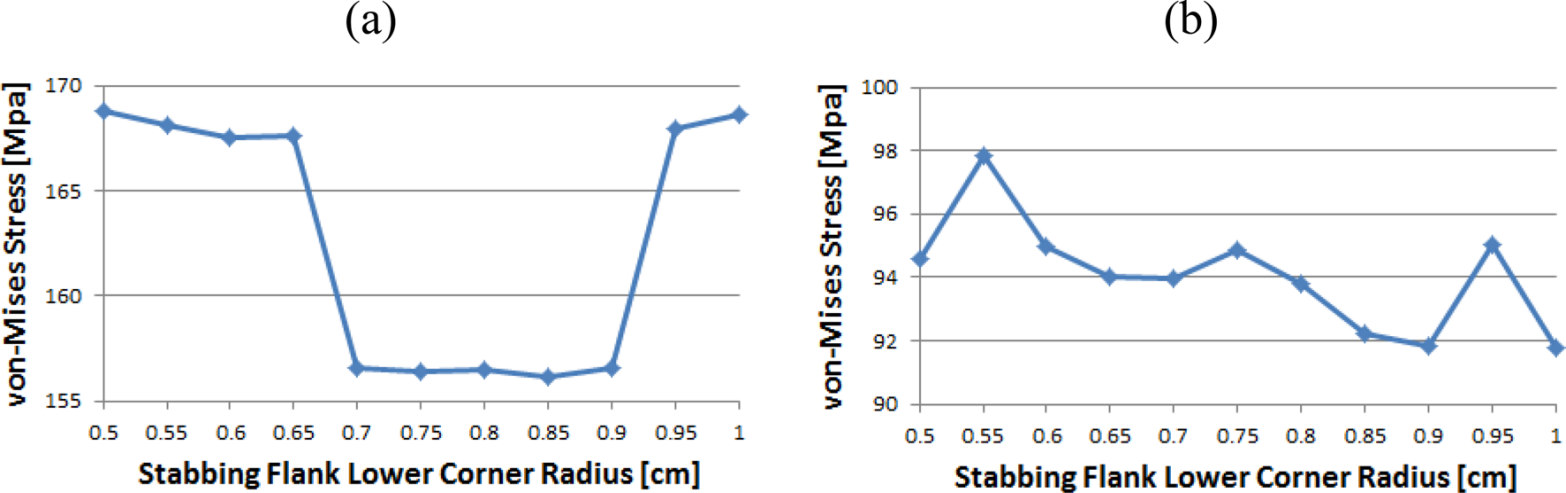
Stress change trend with lower corner radius of stab flank: (**a**) loading flank peak, (**b**) loading flank base of coupling thread 1 (coupling LET).

In the case of the loading flank peak, as shown in Fig. 15, the value decreased continuously without a significant difference from the outset, and when it reached 0.7, it approached 156 MPa. At 0.95, the value increased significantly. The maximum and minimum values are seen at 0.5 and 0.85, respectively. In the case of the loading flank base, the values are in the range of 92–98 MPa. The maximum and minimum values appear at 0.55 and 1, respectively.

### Coupling thread 2

**Figure 16:**
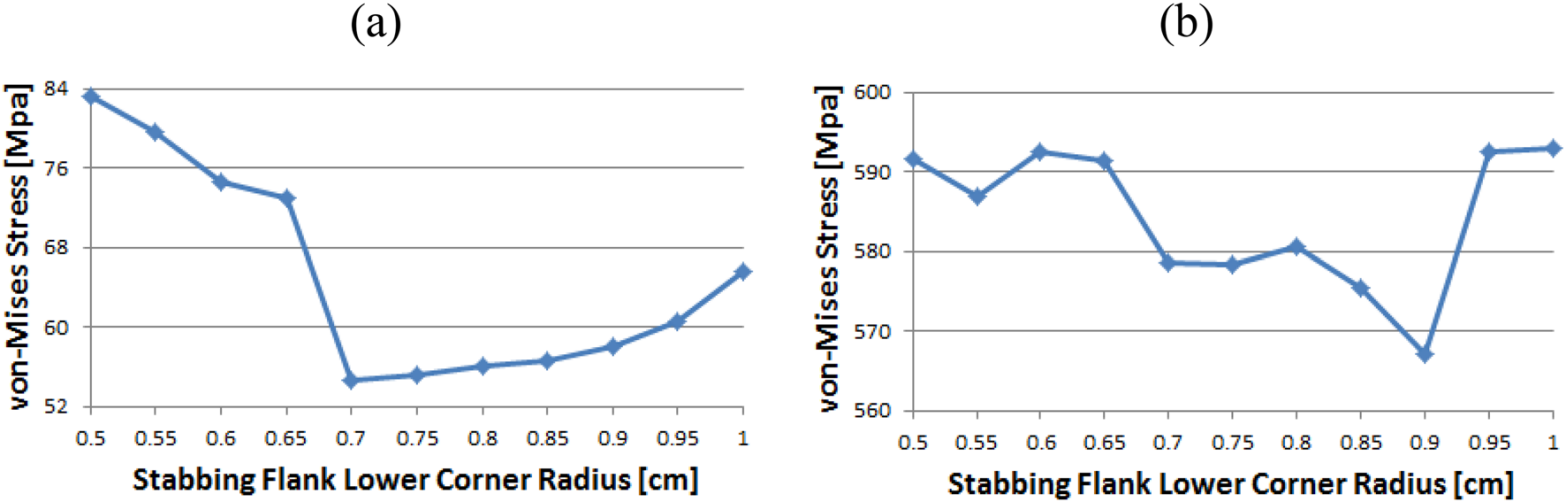
Stress change trend with lower corner radius of stab flank: (**a**) stab flank base, (**b**) stab flank peak of coupling thread 2.

Figure 16 represents the case of the stab flank base and it is discovered that the stress values are diminished as the corner radius increased. The value shows the minimum at 0.7 and then it is gradually increased as the radius increases from 0.7 to 1. The lowest value can be found at 0.7 and the highest value at 0.5, respectively. When the corner radius is altered for a stab flank peak, the von Mises stress is as a rule decreased from 0.5 to 0.9. At 0.9, it is at its minimum value and then the stress is increased thereafter. The maximum value is at 1.

**Figure 17:**
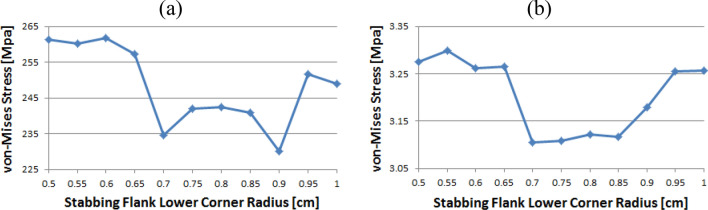
Stress change trend with lower corner radius of stab flank: (**a**) stab flank base, (**b**) stab flank peak of coupling thread 2.

At first glance, as shown in Fig. 17, it appears that the stress at the loading flank base steadily decreases up to 0.7. The von Mises stress slightly changes from 0.7 to 0.9, where it reaches its minimum value at 0.9. Subsequently, it increases and then decreases. The maximum value is observed at 0.5. In the case of the loading flank peak, the stress initially increased before continuously decreasing to a minimum at 0.7. The value increased continuously from 0.7 to 1. The maximum value is at 0.55; however, this difference is not statistically significant.

**Figure 18:**
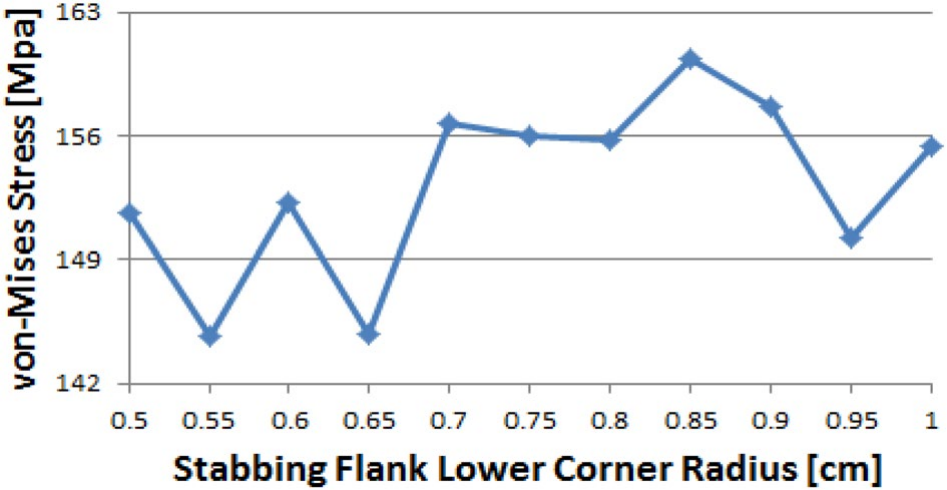
Stress change trend with lower corner radius of stab flank: loading flank peak (pin base) of coupling thread 2.

In the case of the loading flank peak (Pin Base), as shown in Fig. 18, the value is initially decreased from 0.5 to 0.55. Subsequently, it repeatedly increased and decreased. The maximum measured value is at 0.85.

### Coupling thread 3

**Figure 19:**
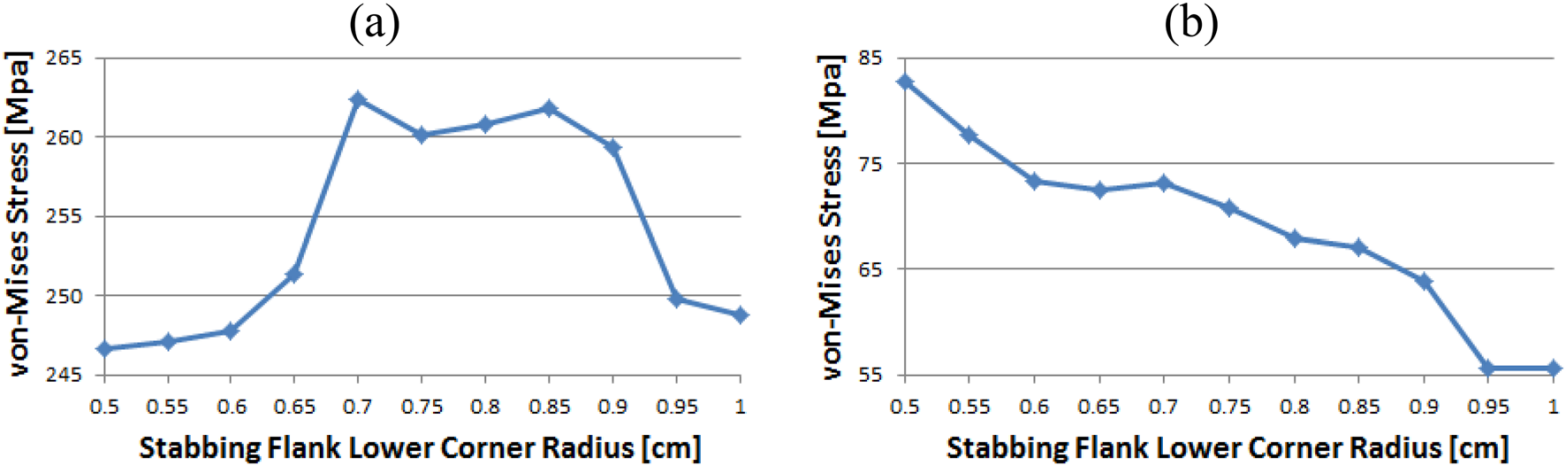
Stress change trend with lower corner radius of stab flank: (**a**) stab flank peak, (**b**) stab flank base of coupling thread 3.

As shown in Fig. 19, the value of the stab flank peak increased continuously from 0.5 to a maximum of 0.7. Subsequently, the value decreased to 0.85. It is confirmed that the value continued to decrease 0.85 onwards. The minimum stress is at 0.5. In the case of the stab flank base, the value decreased, with the exception of the 0.7 corner radius. The minimum and maximum values are at 1 and 0.5, respectively.

### Pin thread 2

**Figure 20:**
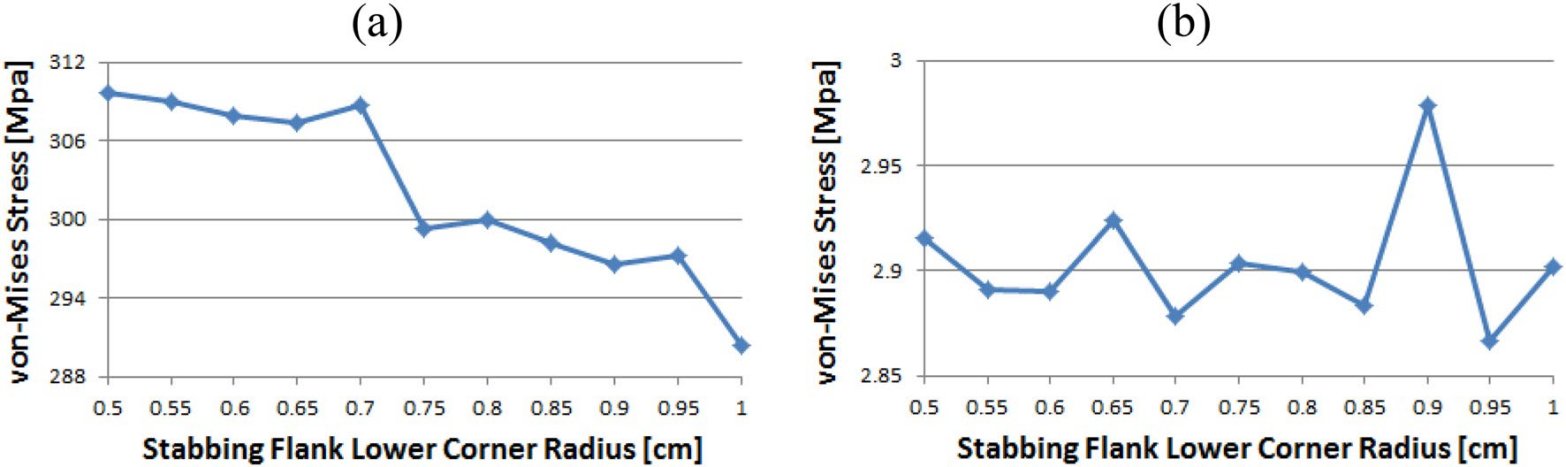
Stress change trend with lower corner radius of stab flank: (**a**) loading flank peak (pin base), (**b**) loading flank peak of pin thread 2.

When the corner radii are 0.7, 0.8, or 0.95, the value of the loading flank peak (pin base) is slightly increased, as shown in Fig. 20. Overall, these values continued to decline. The minimum and maximum values are at 1 and 0.5, respectively. At the loading flank peak of pin thread 2, the stress repeatedly decreased and increased. Maximum value is determined to be at 0.9, while minimum value is at 0.95.

**Figure 21:**
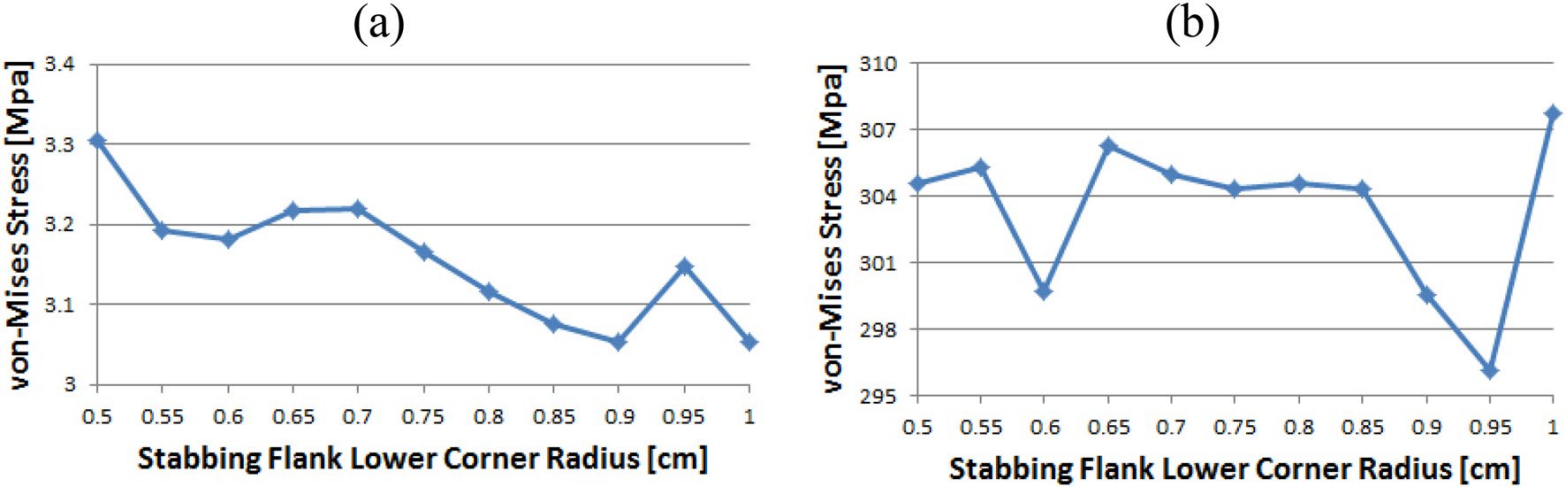
Stress change trend with lower corner radius of stab flank: (**a**) loading flank base (pin peak), (**b**) loading flank base pin thread 2.

Figure 21 shows the case of the loading flank base (pin peak). The value is increased from 0.6 to 0.7 and 0.95, while it is discovered that the stress is decreased with other corner radii. The maximum and minimum values are at 0.5 and 1, respectively. In the case of the loading flank base pin thread 2, there are abrupt stress spikes at 0.6 and 0.95. The value is decreasing at other corner radii, and its minimum value is found at 0.95 and the maximum value is found at 1.

**Figure 22:**
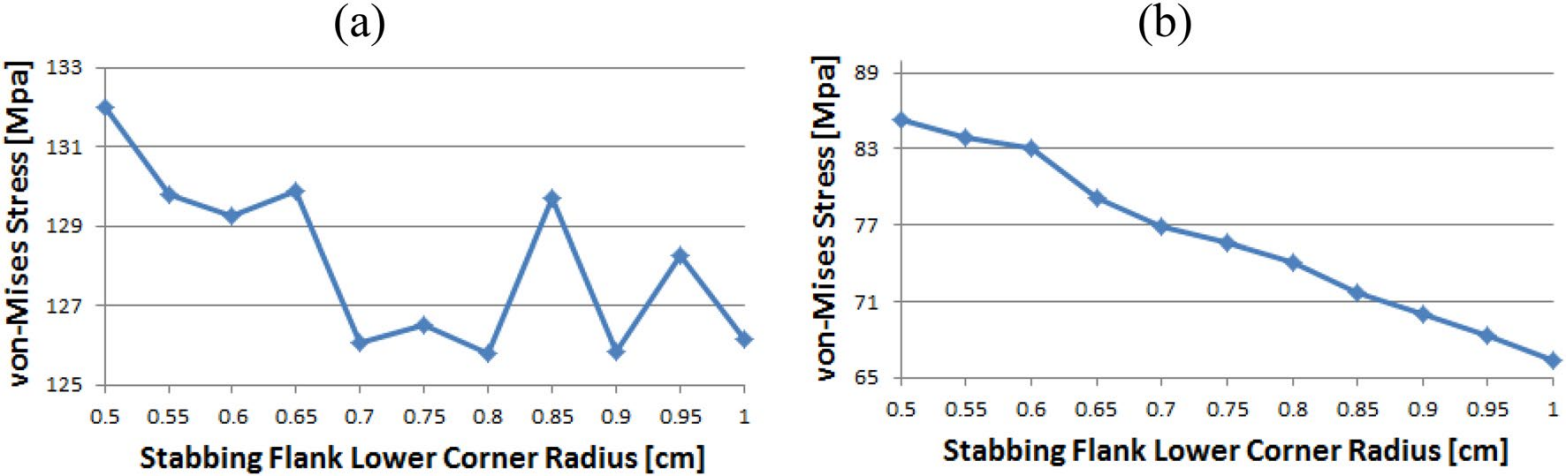
Stress change trend with lower corner radius of stab flank: (**a**) stab flank peak, (**b**) stab flank base of pin thread 2.

In the case of the stab flank peak, as shown in Fig. 22, the stress is initially reduced up to the case of 0.6 and then it is fluctuated repeatedly. The maximum value is at 0.5, while the minimum is at 0.8. As the corner radius increases, the value of the stab flank base of pin thread 2 decreases continuously. The maximum and minimum values are at 0.5 and 1, respectively.

### Pin thread 1 (pin LET)

**Figure 23:**
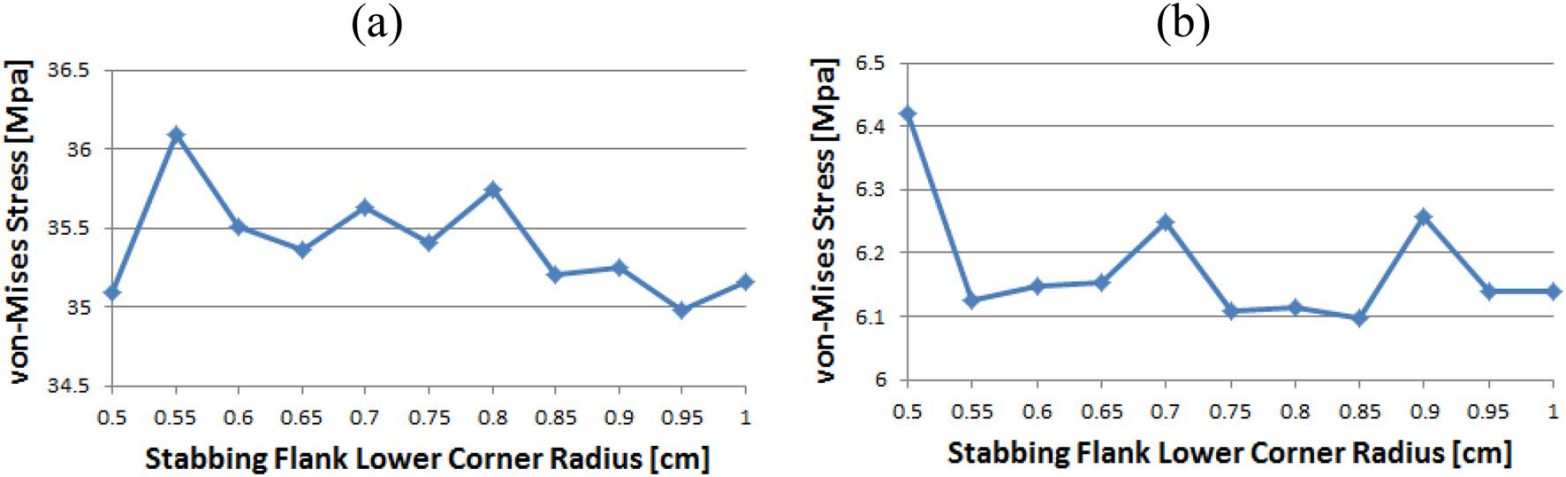
Stress change trend with lower corner radius of stab flank: (**a**) loading flank peak (pin base), (**b**) loading flank peak of pin thread 1 (pin LET).

In the case of the loading flank peak (pin base), as shown in Fig. 23, it is observed that the value is initially increased at 0.55 and subsequently it is repeatedly increased and then decreased; however, there is no discernible difference. The maximum value is at 0.55 and minimum is at 0.95. In the case of the loading flank peak, the stress values are between 6.1 and 6.2 MPa, with the exception of the corner radius values of 0.5, 0.7, and 0.9. The maximum value is determined to be 0.5, while the minimum value is at 0.85. There is an insignificant difference between these values.

**Figure 24:**
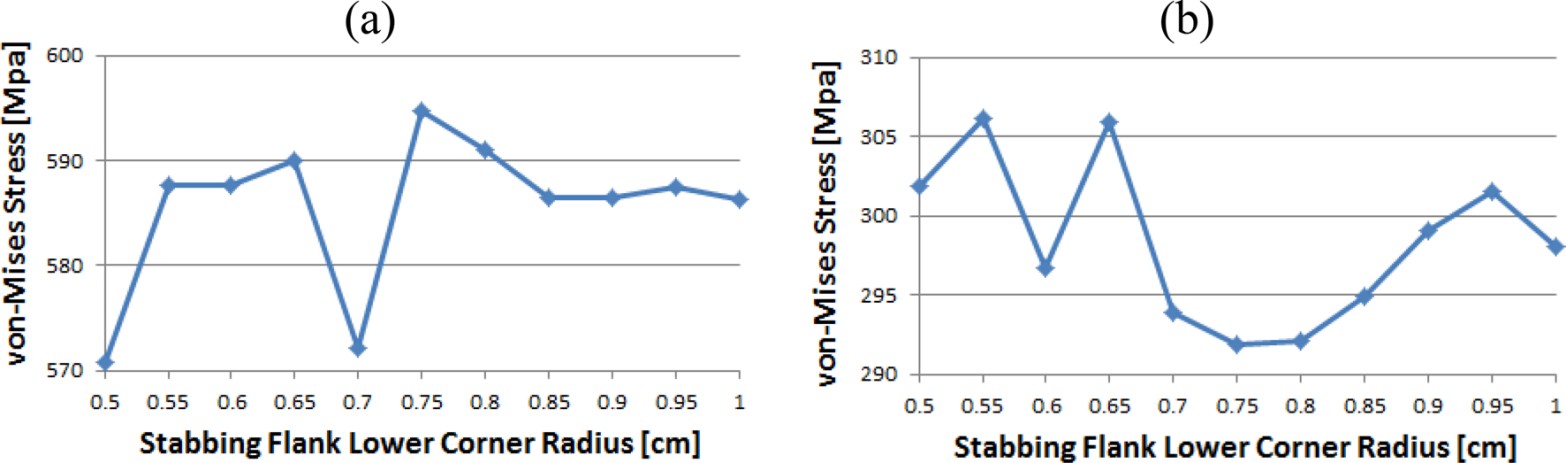
Stress change trend with lower corner radius of stab flank: (**a**) loading flank base, (**b**) stab flank peak of pin thread 1 (pin LET).

Figure 24 presents the result of the loading flank base; the stress is increased from 0.5 to 0.65 before plummeting at 0.7. The value is then increased to the maximum at 0.75 before decreasing. The maximum value is at 0.75, and minimum is at 0.5. In the case of stab flank peak, the stress is at first increased and then decreased on multiple occasions. It is confirmed that the value is increased continuously from 0.75 to 0.95 and decreased once more at 1. The maximum and minimum values are at 0.55 and 0.75, respectively.

**Figure 25:**
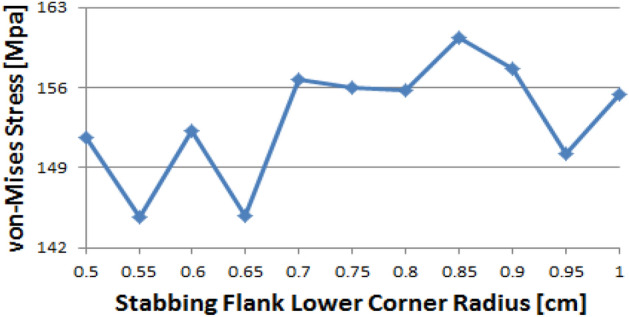
Stress change trend with lower corner radius of stab flank: stab flank base of pin thread 1 (pin LET).

Figure 25 shows that the stress value of the stab flank base is fluctuated and the maximum and minimum values are at 0.85 and at 0.55, respectively.

## Results

As there is no clear pattern in the data in the preceding section, it is difficult to identify the ideal radius value. To ascertain the ideal value, a complicated study incorporating the maximum and root-mean-square (RMS) values is necessary. The RMS value is defined as $\sqrt{1/n{\sum }_{k=1}^{n}{x}_{k}^{2}}$, which is a representative value for the number sequences.

According to Table 2, the loading flank base section with 0.75 cm lower corner radius has the maximum stress value at pin thread 1 (pin LET). The stab flank base's bottom corner radii of 0.50 cm and 1.00 cm have the highest von Mises stress differential. The stress levels of the other components did not differ significantly. In the case of the bottom corner radius of 0.65 cm, the RMS stress is at its maximum; in the case when it is 0.70 cm, it is at its minimum.

**Table 2:** Stress values at each part and RMS with different stab flank lower corner radius: pin thread 1 (pin LET).

Radius/part (cm)	Loading flank base (MPa)	Loading flank peak (pin base) (MPa)	Loading flank peak (MPa)	Stab flank peak (MPa)	Stab flank base (MPa)	RMS (MPa)
0.50	**570.77**	35.10	6.42	301.84	**140.09**	295.90
0.55	587.64	36.09	6.13	306.09	135.53	302.89
0.60	587.70	35.51	6.15	296.69	130.90	300.62
0.65	590.02	35.36	6.15	305.93	129.37	**303.23**
0.70	572.10	35.63	6.25	293.90	125.79	**293.53**
0.75	**594.77**	35.40	6.11	291.90	121.63	301.68
0.80	591.04	35.74	6.11	292.14	120.10	300.14
0.85	586.40	35.21	6.10	294.90	117.53	298.64
0.90	586.52	35.25	6.26	299.06	113.88	299.23
0.95	587.44	34.98	6.14	301.53	113.16	300.02
1.00	586.31	35.15	6.14	298.07	**110.90**	298.72

Significant values are in [bold]

From Table 3, the maximum stress for pin thread 2 is observed in the loading flank peak (pin base) region of the 0.50 cm lower corner radius, and the lowest value is also observed there. At the loading flank peak (pin base), where the radius at the lower corner is in the range 0.50–1.00 cm, the largest stress gap is seen. The von Mises stress values in the other sections did not differ significantly. The lower corner radius is measured with a minimum stress at 0.95 cm and maximum stress at 0.50 cm to achieve the RMS values.

**Table 3:** Stress values at each part and RMS with different stab flank lower corner radius: pin thread 2.

Radius/part (cm)	Loading flank base (pin peak) (MPa)	Loading flank base (MPa)	Loading flank peak (pin base) (MPa)	Loading flank peak (MPa)	Stab flank peak (MPa)	Stab flank base (MPa)	RMS (MPa)
0.50	3.30	304.56	**309.67**	2.92	132.01	85.28	**188.58**
0.55	3.19	305.31	309.04	2.89	129.77	83.94	188.25
0.60	3.18	299.68	307.89	2.89	129.26	83.11	186.30
0.65	3.22	306.29	307.36	2.92	129.89	79.17	187.72
0.70	3.22	304.99	308.76	2.88	126.05	76.91	187.16
0.75	3.17	304.38	299.28	2.90	126.53	75.64	184.38
0.80	3.12	304.56	299.89	2.90	125.80	74.08	184.40
0.85	3.08	304.36	298.22	2.88	129.70	71.65	184.18
0.90	3.05	299.59	296.56	2.98	125.86	69.95	181.87
0.95	3.15	296.13	297.32	2.87	128.26	68.30	**181.30**
1.00	3.05	307.73	**290.33**	2.90	126.17	66.29	182.26

Significant values are in [bold]

According to Table 4, the maximum value for the case of Coupling Thread 1 (coupling LET) is in the loading flank peak (pin base) section with the lower corner radius of 0.65 cm and minimum value with the lower corner radius of 0.85 cm. At the loading flank peak (pin base), the lower corner radii of 0.65 cm and 0.85 cm showed the highest difference for the stress values. The stress values did not vary significantly in the other areas. According to the RMS results, the maximum and minimum values are 0.65 and 0.85 cm, respectively.

**Table 4:** Stress values at each part and RMS with different stab flank lower corner radius: coupling thread 1 (Coupling LET).

Radius/part (cm)	Loading flank peak (MPa)	Loading flank peak (pin base) (MPa)	Loading flank peak 2 (MPa)	Loading flank base (MPa)	RMS (MPa)
0.50	168.77	174.87	34.96	94.57	131.56
0.55	168.09	175.80	34.67	97.87	132.23
0.60	167.51	176.15	34.61	95.01	131.64
0.65	167.60	**179.99**	34.79	94.00	**132.79**
0.70	156.57	175.52	30.06	93.97	127.53
0.75	156.38	175.00	30.33	94.88	127.48
0.80	156.48	175.01	30.22	93.80	127.30
0.85	156.15	**167.16**	30.55	92.25	**124.27**
0.90	156.59	175.03	29.97	91.82	126.97
0.95	167.95	175.28	34.73	95.04	131.50
1.00	168.60	176.75	35.23	91.77	131.65

Significant values are in [bold]

Table 5 shows that the part of the stab flank peak with the lower corner radius of 0.60 cm had the highest value for Coupling Thread 2 at that location. The largest von Mises stress difference is observed at the lower corner radii of 0.60 cm and 0.90 cm. The stress at the other components did not vary much. For the lower corner radius, the RMS findings are obtained with a maximum at 0.60 cm and minimum at 0.90 cm.

**Table 5:** Stress values at each part and RMS with different stab flank lower corner radius: coupling thread 2.

Radius/part (cm)	Stab flank base (MPa)	Stab flank peak (MPa)	Loading flank peak (pin base) (MPa)	Loading flank peak (MPa)	Loading flank base (MPa)	RMS (MPa)
0.50	83.21	591.69	151.59	3.27	261.42	299.45
0.55	79.69	586.92	144.69	3.30	260.25	296.48
0.60	74.64	**592.50**	152.25	3.26	**261.86**	**299.46**
0.65	72.96	591.29	144.78	3.27	257.33	297.37
0.70	54.67	578.57	156.65	3.11	234.64	288.91
0.75	55.13	578.39	156.04	3.11	241.96	289.99
0.80	56.03	580.59	155.76	3.12	242.56	290.97
0.85	56.64	575.52	160.34	3.12	240.80	289.18
0.90	58.13	**567.08**	157.67	3.18	**230.10**	**283.82**
0.95	60.65	592.57	150.21	3.26	251.76	296.91
1.00	65.58	592.97	155.39	3.26	248.96	297.34

Significant values are in [bold]

According to Table 6, coupling thread 3 had the maximum value at 0.70 cm of the lower corner radius for the stab flank peak region and minimum value at 0.50 cm of the lower corner radius. The stab flank peak's bottom corner radius, between 0.50 and 0.70 cm, is where the largest stress differential is found. Stress values are not considerably different in the remaining stab flank base portions. For the lower corner radius, the RMS findings are obtained with a maximum of 0.70 cm and minimum of 1.00 cm.

**Table 6:** Stress values at each part and RMS with different stab flank lower corner radius: coupling thread 3.

Radius/part (cm)	Stab flank base (MPa)	Stab flank peak (MPa)	RMS (Mpa)
0.50	82.70	**246.66**	183.96
0.55	77.66	247.09	183.15
0.60	73.39	247.82	182.76
0.65	72.50	251.38	185.00
0.70	73.12	**262.34**	**192.57**
0.75	70.73	260.10	190.60
0.80	67.84	260.82	190.56
0.85	67.10	261.78	191.09
0.90	63.95	259.36	188.89
0.95	55.61	249.81	180.97
1.00	55.54	248.81	**180.27**

Significant values are in [bold]

In Table 7, the final calculated RMS values are listed using the RMS values for each thread after each thread-by-thread examination. The lowest corner radius of pin thread 1 has a maximum RMS value of 0.65 cm. The difference between the RMS values is greatest for the lower corner radius, between 0.60 and 0.90 cm, at coupling thread 2. The final RMS results show a maximum at 0.65 cm and minimum at 0.90 cm.

**Table 7:** Final RMS value comparison at each thread.

Radius/thread (cm)	RMS value (MPa)					RMS (MPa)
	Pin thread 1	Pin thread 2	Coupling thread 1	Coupling thread 2	Coupling thread 3	
0.50	295.90	188.58	131.56	299.45	183.96	229.75
0.55	302.89	188.25	132.23	296.48	183.15	230.70
0.60	300.62	186.30	131.64	**299.46**	182.76	230.43
0.65	**303.23**	187.72	132.79	297.37	185.00	**231.29**
0.70	**293.53**	187.16	127.53	288.91	192.57	227.16
0.75	301.68	184.38	127.48	289.99	190.60	228.77
0.80	300.14	184.40	127.30	290.97	190.56	228.59
0.85	298.64	184.18	124.27	289.18	191.09	227.46
0.90	299.23	181.87	126.97	**283.82**	188.89	**225.82**
0.95	300.02	181.30	131.50	296.91	180.97	228.51
1.00	298.72	182.26	131.65	297.34	180.27	228.34

Significant values are in [bold]

In order to determine the optimized value, a statistic selection method is proposed. For all the part of 5 thread cases (pin thread 1, 2 and coupling thread 1, 2, 3), the lower corner radii are ranked based on the stress value to select top three and bottom three cases, respectively. And then, the frequency of the radii at the minimum rank of 1 is counted and the case with the most counts is determined as optimal. Table 8 shows that the lower corner radii of 0.90 cm and 1.00 cm occurred six times at Rank 1 in minimum. However, the lower corner radius of 0.90 cm occurred only once at Rank 1 in maximum, while the lower corner radius of 1.00 cm occurred two times in the same category. Thus, the lower corner radius of 0.90 cm is chosen to be the best value.

**Table 8:** Optimized value selection by stress size ranking.

Thread	Part	Radius (cm)					
		Minimum (rank)			Maximum (rank)		
		1	2	3	1	2	3
Pin thread 1	Loading flank base	0.50	0.70	1.00	0.75	0.80	0.65
	Loading flank peak (pin base)	0.95	0.50	1.00	0.55	0.80	0.70
	Loading flank peak	0.85	0.75	0.80	0.50	0.90	0.70
	Stab flank peak	0.75	0.80	0.70	0.55	0.65	0.50
	Stab flank base	1.00	0.95	0.90	0.50	0.55	0.60
	Coupling thread 21(RMS)	0.70	0.50	0.85	0.65	0.55	0.75
Pin thread 2	Loading flank base (pin peak)	0.90	1.00	0.85	0.50	0.70	0.65
	Loading flank base	0.95	0.90	0.60	1.00	0.65	0.55
	Loading flank peak (pin base)	1.00	0.90	0.95	0.50	0.55	0.70
	Loading flank peak	0.95	0.70	0.85	0.90	0.65	0.50
	Stab flank peak	0.80	0.90	0.70	0.50	0.65	0.55
	Stab flank base	1.00	0.95	0.90	0.50	0.55	0.60
	Coupling thread 20(RMS)	0.95	0.90	1.00	0.50	0.55	0.65
Coupling thread 1	Loading flank peak	0.85	0.75	0.80	0.50	1.00	0.55
	Loading flank peak(pin base)	0.85	0.50	0.75	0.65	1.00	0.60
	Loading flank peak 2	0.90	0.70	0.80	1.00	0.50	0.65
	Loading flank base	1.00	0.90	0.85	0.55	0.95	0.60
	Coupling thread 1(RMS)	0.85	0.90	0.80	0.65	0.55	1.00
Coupling thread 2	Stab flank base	0.70	0.75	0.80	0.50	0.55	0.60
	Stab flank peak	0.90	0.85	0.75	1.00	0.95	0.60
	Loading flank peak(pin base)	0.55	0.65	0.95	0.85	0.90	0.70
	Loading flank peak	0.70	0.75	0.85	0.55	0.50	0.65
	Loading flank base	0.90	0.70	0.85	0.60	0.50	0.55
	Coupling thread 2(RMS)	0.90	0.70	0.85	0.60	0.50	0.65
Coupling thread 3	Stab flank base	1.00	0.95	0.90	0.50	0.55	0.60
	Stab flank peak	0.50	0.55	0.60	0.70	0.85	0.80
	Coupling thread 3(RMS)	1.00	0.95	0.60	0.70	0.85	0.75
Total RMS		0.90	0.70	0.85	0.65	0.55	0.60

**Figure 26:**
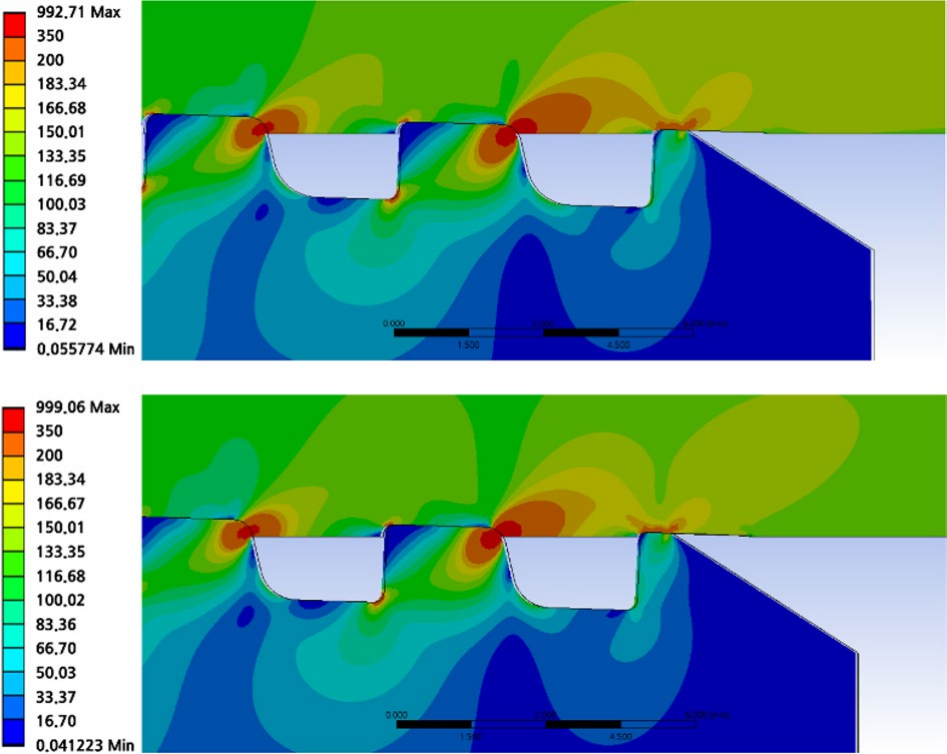
Stress comparison with lower corner radius of 0.76 cm (up) and 0.9 cm (down) (coupling LET portion).

**Figure 27:**
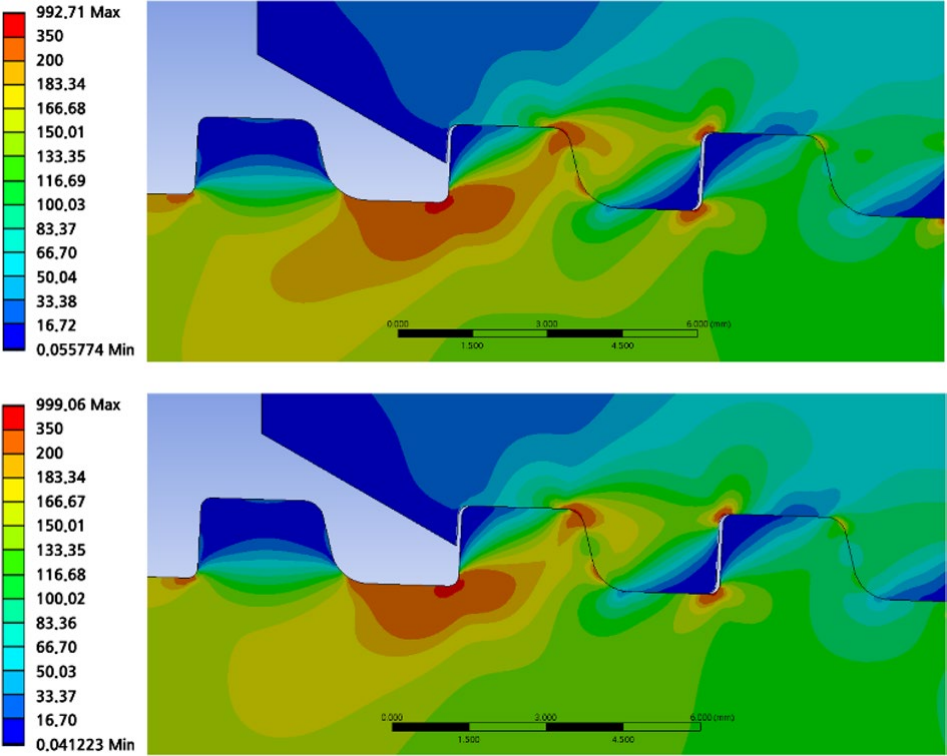
Stress comparison with lower corner radius of 0.76 cm (up) and 0.9 cm (down) (pin LET portion).

Now, using computer-assisted engineering, the axial stress is compared with the original (0.76 cm) and optimized (0.9 cm) values of the lower corner radius. Table 9 summarizes the maximum von Mises stress for each thread. The stress distribution is comparable, as shown in Figs. 26 and 27; however, the stress value at the lower corner radius of 0.9 cm is lower than that in the case of 0.76 cm, and the same holds true for the loading flank base of the pin thread 1, stab flank peak, loading flank base, loading flank peak (pin base), loading flank peak of coupling thread 1, loading flank peak of coupling thread 2, loading flank base, and stab flank peak of coupling thread 3.

**Table 9:** Stress comparison with 0.76 cm (original value) and 0.9 cm (optimal value) of lower corner radius.

Thread	Part/radius	0.76 cm (MPa)	0.90 cm (MPa)	Difference
Pin thread 1	Loading flank base	594.77	586.52	8.25(1%↓)
	Stab flank peak	291.90	299.06	**7.16 (3%↑)**
	Pin thread 1 (RMS)	301.68	299.23	2.45 (0.8%↓)
Pin thread 2	Loading flank base	304.38	299.59	4.79 (2%↓)
	Loading flank peak (pin base)	299.28	296.56	2.72 (1%↓)
	Pin thread 2 (RMS)	184.38	181.87	2.51 (1%↓)
Coupling thread 1	Loading flank peak	156.38	156.59	**0.21 (0.1%↑)**
	Coupling thread 1 (RMS)	127.48	126.97	0.51 (0.4%↓)
Coupling thread 2	Stab flank peak	578.39	567.08	11.31 (2%↓)
	Loading flank base	241.96	230.10	**11.86 (5%↓)**
	Coupling thread 2 (RMS)	289.99	283.82	6.17 (2%↓)
Coupling thread 3	Stab flank peak	260.10	259.36	0.74 (0.3%↓)
	Coupling thread 3 (RMS)	190.60	188.89	1.71 (1%↓)
RMS		228.77	225.82	2.95 (1%↓)

Significant values are in [bold]

To acquire the final result, lower corner radius of 0.76 and 0.90 cm are chosen as the stressed areas, and the RMS values are matched. The comparison between the lower corner radius of 0.76 cm (original value) and 0.9 cm (optimal value) is as follows. From Table 9, it can be seen that the stress value increased with the optimal value of the lower corner radius compared to the original case at the loading flank base of coupling thread 1 and slab flank peak of pin thread 1. However, the stresses decreased throughout the remainder, and the stress value at the loading flank base of coupling thread 2 decreased by 5%. It is also verified that the number of parts decreased. Finally, the ultimate RMS value decreased by 1%.

## Conclusions

This study enhances the premium casing connection used to extract the shale gas and hydrocarbons from the deep reserves, particularly for the stab flank. When an axial (either compressive or tensile) force is applied to the premium casing system based on the dimensional change for optimization, the tendency of the von Mises stress distribution of each weak component, as well as the stress and RMS of each component, is thoroughly determined.

The bottom corner radius of the stab flank is improved to 0.9 cm based on the parametric studies of the buttress type API-based premium casing connection system. According to the computational analysis of the distributed stress under the tensile and compression forces according to the variation in the lower corner radius, the axial stress on the stab flank bases of each screw decreased when the lower corner radii changed. In this study, a new high-end connection system with exceptional vibration resistance and durability is developed. It is possible to streamline the design to lower the stresses, thereby enabling the development of premium connections, which are more resilient to vibration-type loading. This makes it possible to construct a suitable premium connection based on the needs, efficiency, and economics worldwide.

The contribution of this study can be summarized in the following two points: (1) it is possible to design a premium connection system for shale gas drilling with excellent performance in terms of stress through a parametric study related to stab flank, (2) a new technique is proposed to rank each part of threaded connection through stress distribution and statistically analyze it to find optimal parameters.

However, solely controlling the parameters linked to the stab flank may have a detrimental effect on other thread locations, such as the loading flank, which should also be optimized. Further experiments are required to confirm these findings. Future research is expected to evaluate the von Mises stress changes when additional factors, such as thread incision angle, length, pitch, and space length vary, as well as the contact stress analysis for improving gas sealing capability. In addition, the bending stress should be considered with a series of 3D simulation to completely validate the structural durability of the premium connection system.

## Data Availability

The datasets used and/or analysed during the current study available from the corresponding author on reasonable request.
